# Brain White-Matter Degeneration Due to Aging and Parkinson Disease as Revealed by Double Diffusion Encoding

**DOI:** 10.3389/fnins.2020.584510

**Published:** 2020-10-15

**Authors:** Kouhei Kamiya, Koji Kamagata, Kotaro Ogaki, Taku Hatano, Takashi Ogawa, Haruka Takeshige-Amano, Syo Murata, Christina Andica, Katsutoshi Murata, Thorsten Feiweier, Masaaki Hori, Nobutaka Hattori, Shigeki Aoki

**Affiliations:** ^1^Department of Radiology, Juntendo University School of Medicine, Tokyo, Japan; ^2^Department of Radiology, Toho University, Tokyo, Japan; ^3^Department of Neurology, Juntendo University School of Medicine, Tokyo, Japan; ^4^Siemens Healthcare K.K., Tokyo, Japan; ^5^Siemens Healthcare GmbH, Erlangen, Germany

**Keywords:** aging, Parkinson disease, diffusion MRI, double diffusion encoding, microstructure

## Abstract

Microstructure imaging by means of multidimensional diffusion encoding is increasingly applied in clinical research, with expectations that it yields a parameter that better correlates with clinical disability than current methods based on single diffusion encoding. Under the assumption that diffusion within a voxel can be well described by a collection of diffusion tensors, several parameters of this diffusion tensor distribution can be derived, including mean size, variance of sizes, orientational dispersion, and microscopic anisotropy. The information provided by multidimensional diffusion encoding also enables us to decompose the sources of the conventional fractional anisotropy and mean kurtosis. In this study, we explored the utility of the diffusion tensor distribution approach for characterizing white-matter degeneration in aging and in Parkinson disease by using double diffusion encoding. Data from 23 healthy older subjects and 27 patients with Parkinson disease were analyzed. Advanced age was associated with greater mean size and size variances, as well as smaller microscopic anisotropy. By analyzing the parameters underlying diffusion kurtosis, we found that the reductions of kurtosis in aging and Parkinson disease reported in the literature are likely driven by the reduction in microscopic anisotropy. Furthermore, microscopic anisotropy correlated with the severity of motor impairment in the patients with Parkinson disease. The present results support the use of multidimensional diffusion encoding in clinical studies and are encouraging for its future clinical implementation.

## Introduction

Parkinson disease (PD) is a neurodegenerative disorder characterized by motor symptoms (akinesia, resting tremor, and rigidity) and a wide range of cognitive, neuropsychiatric, and autonomic dysfunctions (Poewe et al., 2017). Advanced age is a major risk factor for the development of PD and is also associated with faster motor decline (Levy, 2007; Collier et al., 2017). Pathologically, PD is characterized by widespread aggregation of α-synuclein-immunoreactive inclusions in the form of Lewy pathology within both the neuronal cytoplasm (Lewy bodies) and axons (Lewy neurites) (Braak et al., 2003; Kanazawa et al., 2012; Poewe et al., 2017). Neuropathological studies have indicated that Lewy pathologies evolve along major fiber pathways, beginning in the brain stem and eventually advancing to the neocortical regions (Braak et al., 2003). Accumulating evidence has suggested that axonal degeneration is an early event in the process of neurodegeneration that is common to PD and other age-related neurological diseases (Kurowska et al., 2016; Salvadores et al., 2017). Non-invasive characterization of the neurodegeneration underlying the pathogenesis and progression of PD is of high clinical demand, because it will aid in the development of novel therapeutic strategies and in monitoring the effects of treatment.

Diffusion MRI (dMRI) is uniquely sensitive to tissue features on the micrometer scale and therefore has been widely used to study neurodegeneration in aging (Madden et al., 2012; Coutu et al., 2014; Billiet et al., 2015; Benitez et al., 2018; Guerreri et al., 2019) and diseases (Goveas et al., 2015; Andica et al., 2019b). In PD, the majority of studies have applied diffusion tensor imaging (DTI) (Basser, 1995) and have consistently reported smaller fractional anisotropy (FA) and greater mean diffusivity (MD) in the white matter of patients than of controls (for a recent review and meta-analysis see Atkinson-Clement et al., 2017). Several groups (Wang et al., 2011; Kamagata et al., 2013, 2014; Surova et al., 2016, 2018) further explored the utility of dMRI acquisition with higher b-values than used in DTI, which is typically analyzed by means of diffusion kurtosis imaging (DKI) (Jensen et al., 2005). The works by Kamagata et al. (2013, 2014) showed that kurtosis in the white matter is reduced in patients and that DKI is more sensitive to white-matter degeneration than is DTI. Of note, several studies demonstrated that white-matter degeneration as detected by dMRI precedes macroscopic gray matter atrophy (Agosta et al., 2013; Duncan et al., 2016; Rektor et al., 2018), suggesting the potential of dMRI parameters as early biomarkers of PD.

Regarding acquisition, both DTI and DKI use single diffusion encoding (SDE), which uses one pair of diffusion-sensitizing gradients. Multidimensional diffusion encoding (Mitra, 1995; Topgaard, 2017), that can be realized by using either double diffusion encoding (DDE) (Cory et al., 1990; Callaghan and Xia, 1991), triple diffusion encoding (Mori and Van Zijl, 1995), or continuous gradient waveforms (Caprihan et al., 1996; Callaghan, 1997; Eriksson et al., 2013), has recently gained attention in clinical studies because these methods provide more specific information about the tissue microstructure. For example, cumulant expansion of the DDE signal up to the fourth-order term of the gradient amplitude (Jespersen, 2012) shows DDE provides unique information that is not contained in the standard diffusion and kurtosis tensors available with SDE. In addition to DTI/DKI parameters, at least two new properties of biological interest can be obtained: the microscopic anisotropy (Cory et al., 1990; Cheng and Cory, 1999; Callaghan and Komlosh, 2002; Özarslan and Basser, 2008; Shemesh et al., 2009; Lawrenz et al., 2010; Jespersen et al., 2013; Lasič et al., 2014) and the variance of isotropic diffusivities among individual microenvironments (compartments) (Szczepankiewicz et al., 2016; Westin et al., 2016). Several works have demonstrated that the microscopic anisotropy, a measure of diffusion anisotropy that is not confounded by orientational dispersion of axons or fibers, can be estimated reliably in nervous tissues (Komlosh et al., 2007, 2008; Shemesh and Cohen, 2011; Shemesh et al., 2012; Jespersen et al., 2013; Lawrenz and Finsterbusch, 2013; Lawrenz et al., 2015). Microscopic anisotropy has been shown to be useful for characterizing white-matter degeneration in aging (Lawrenz et al., 2016) and in multiple sclerosis (Yang et al., 2018; Andersen et al., 2020). The variance of isotropic diffusivities has also been suggested to be promising for deducing the microstructural underpinnings of the diffusion changes in diseases like schizophrenia (Westin et al., 2016). Moreover, multidimensional diffusion encoding enables us to decompose the sources of DTI/DKI parameters (Lasič et al., 2014; Szczepankiewicz et al., 2016; Westin et al., 2016; Henriques et al., 2020). Such analyses are expected to improve our understanding of neurodegeneration and the sensitivity of imaging to pathology. For example, if the pathology affects two underlying sources of a particular DTI/DKI parameter in opposite directions, as has been suggested in Douaud et al. (2011); Lawrenz et al. (2016), Lampinen et al. (2019), the sensitivity and interpretability of that parameter would be limited, whereas separation of each source may provide useful information.

In this study, we analyzed DDE data by using the covariance tensor framework (Westin et al., 2016) that works under the diffusion tensor distribution (DTD) model (Jian et al., 2007). Given the DTD model, continuous waveforms would offer more efficient diffusion weighting and faster acquisition than DDE (Nilsson et al., 2020). We adopted DDE here because this study was planned before the definition of diffusion time for the continuous waveform was made by Lundell et al. (2019). As DTD is a model for the long diffusion time limit where time-dependence is negligible (Novikov et al., 2019), we needed to compare the diffusion time of our measurement with the previous studies (Clark et al., 2001; Portnoy et al., 2013) to rationalize our assumption. The potential limitation arising from neglecting the effects of diffusion time is detailed in “Discussion.”

The aim of this study was to identify the sources of DTI/DKI changes previously reported in aging and PD in terms of the DTD model parameters. We first analyzed correlation with age in healthy older subjects and then examined group differences between the healthy subjects and patients with PD. Finally, we investigated the correlations with the severity of motor impairment in the patients.

## Materials and Methods

### Theory

#### Model and Assumptions

Linking the MRI signal to specific tissue properties involves modeling, simplification of the complex reality relying on a few assumptions. By adopting the DTD model, we assume that the voxel consists of multiple, non-exchanging Gaussian compartments. More precisely, we model the diffusion within a voxel by a distribution of diffusion tensors (free, anisotropic diffusion) (Jian et al., 2007; Topgaard, 2017). In such case, the measurement can be fully characterized by the b-tensor (i.e., b-tensor encoding). In this regard, SDE is linear tensor encoding (LTE), whereas DDE provides planar tensor encoding (PTE) if we apply two diffusion encodings in non-colinear directions (Westin et al., 2016; Topgaard, 2017). Although the DTD model is certainly a crude assumption and not fully validated yet in human brain, describing the tissue by a combination of several diffusion tensors is a common starting point in most dMRI models currently used in clinical studies (Jelescu and Budde, 2017).

Under the DTD model, the signal can be written as:

(1) $$S\left(B\right)=S_{0}\left\langle \text{exp}\left(-\text{B}:{\text{D}}^{c}\right)\right\rangle$$

where *S*(**B**) is the signal, *S*_0_ is the signal without diffusion weighting, **B** is the b-tensor, and **D**^*c*^ is the diffusion tensor of each compartment. The bracket < > denotes the ensemble average over the voxel. The colon denotes a generalized scalar product between the two tensors, **B**:**D** = ∑_*i*_∑_*j*_*B*_*ij*_*D*_*ij*_.

#### Diffusion Parameters

Multidimensional diffusion encoding provides us means to extract summarizing statistics of the distribution of diffusion tensors. Eq. 1 can be expanded (Westin et al., 2016; Topgaard, 2017) to:

(2) $$S\approx S_{0}\text{exp}\left(-\text{B}:\text{D}+\frac{1}{2}\left(\text{B}⊗\text{B}\right):\text{C}\right)$$

where ⊗ denotes a tensor outer product. Here, **D** is the well-known, voxel-averaged diffusion tensor, and **C** is a fourth-order tensor called the covariance tensor. From **D** and **C**, several scalar parameters that summarize microstructural features, including DTI/DKI parameters, can be computed (Westin et al., 2016; Topgaard, 2017; Henriques et al., 2020). Below, we briefly describe the meanings of the parameters used in this study and their relations to each other. First, the DTI parameters MD and FA are defined (Basser, 1995; Westin et al., 2016; Henriques et al., 2019) as:

(3) MD=Tr(D)/3,

(4) $$\text{and}\text{FA}=\sqrt{\frac{3}{2}\frac{V_{\lambda }\left(D\right)}{V_{\lambda }\left(D\right)+{\left(\text{Tr}\left(D\right)/3\right)}^{2}}}$$

*V*_λ_(**D**) denote the variance of eigenvalues of **D**, defined by using the eigenvalues λ_1_, λ_2_, and λ_3_ as Vλ(D)=13∑i=13λi2-(13∑i=13λi)2. MD and FA can be understood as the size and shape of the voxel-averaged diffusion tensor. The definition of μFA has a similar form to that of FA (Westin et al., 2016; Henriques et al., 2019):

(5) $$\mu \text{FA}=\sqrt{\frac{3}{2}\frac{\left\langle V_{\lambda }\left({\text{D}}^{c}\right)\right\rangle }{\left\langle V_{\lambda }\left({\text{D}}^{c}\right)\right\rangle +{\left(\left\langle \text{Tr}\left({\text{D}}^{c}\right)\right\rangle /3\right)}^{2}}}$$

Unlike FA, μFA is not influenced by orientational dispersion and purely reflects the anisotropy (shape) of microstructural environments. In addition, a measure of orientational dispersion (the orientational order parameter, OP) is defined (Lasič et al., 2014; Westin et al., 2016) as:

(6) $${\text{OP}}^{2}=\frac{V_{\lambda }\left(D\right)}{\left\langle V_{\lambda }\left({\text{D}}^{c}\right)\right\rangle }$$

OP equals 1 for perfectly aligned orientations and equals 0 for fully isotropic dispersion. FA is influenced by both μFA and OP (Lasič et al., 2014):

(7) $$\text{FA}=\text{OP}{\left[\mu {\text{FA}}^{-2}+\frac{2}{3}\left({\text{OP}}^{2}-1\right)\right]}^{-1/2}$$

Furthermore, under the DTD assumption, mean kurtosis (MK), as defined in Hansen et al. (2013), can be decomposed into two kurtosis sources (Westin et al., 2016; Henriques et al., 2020):

(8) $$\text{MK}=K_{iso}+K_{aniso}-\psi$$

where

(9) $$K_{iso}=3\frac{V\left(D^{c}\right)}{{\left(\text{Tr}\left(D\right)/3\right)}^{2}}$$

(10) $$\text{and}K_{aniso}=\frac{6}{5}\frac{\left\langle V_{\lambda }\left({\text{D}}^{c}\right)\right\rangle }{{\left(\text{Tr}\left(D\right)/3\right)}^{2}}$$

are the isotropic and anisotropic kurtosis sources. Here, *V*(*D^c^*) is the variance of isotropic diffusivities among compartments (variance of sizes). The last term in Eq. 8 is a factor related to orientational dispersion and can be expressed as (Westin et al., 2016; Henriques et al., 2020)

(11) ψ=25Dxx2+Dyy2+Dzz2+2Dxy2+2Dyz2+2Dzx2(Tr(D)/3)2-65=651(Tr(D)/3)219(2(Dxx2+Dyy2+Dzz2)-2(DxxDyy+DyyDzz+DzzDxx)+6(Dyz2+Dxy2+Dzx2))=65Vλ(D)(Tr(D)/3)2=OP2Kaniso

Using Eq. 11, Eq. 8 can be re-written as:

(12) $$\text{MK}=K_{iso}+\left(1-{\text{OP}}^{2}\right)K_{aniso}$$

In this study, we report MD, FA, MK, *K*_*iso*_, *K*_*aniso*_, μFA, OP, and *V*(*D^c^*). Although the information represented by some parameters overlaps (e.g., both μFA and *K*_*aniso*_ are measures of microscopic anisotropy), having multiple forms of expression helps us to understand how the DTD parameters affect FA and MK (Eqs 7 and 12).

### Participants

This study was carried out in accordance with the Declaration of Helsinki for experiments involving humans. The Institutional Review Board approved this study, and all subjects gave written informed consent prior to participation. Twenty-three healthy older subjects (63.1 ± 7.2 years old) and 27 patients with PD (66.1 ± 6.6 years old) were enrolled. Patients with PD were diagnosed by neurologists on the basis of clinical diagnostic criteria of the Movement Disorder Society (Postuma et al., 2015). Motor function and disease stage were evaluated with the Unified Parkinson’s Disease Rating Scale motor part (UPDRS-III) (Goetz et al., 2008) and the Hoehn and Yahr staging scale (Hoehn and Yahr, 1967). White-matter T2 hyperintensities (WMH) were rated according to the Fazekas scale (Fazekas et al., 1987). The clinical and demographic characteristics of the participants are summarized in Table 1.

**TABLE 1:** Demographic features of the study participants.

	Healthy controls	Patients with PD	P*
Number	23	27	–
Age	63.1 ± 7.2	66.1 ± 6.6	0.14
Sex (male/female)	8/15	16/11	0.08
Disease duration (years)	–	13.4 ± 7.1	–
UPDRS-III	–	13.6 ± 7.7	–
Hoehn–Yahr stage	–	1.7 ± 0.8	–
Levodopa equivalent dose (mg)	–	1040 ± 556	–
Fazekas periventricular white matter	0.57 ± 0.66	0.63 ± 0.69	0.74
Fazekas deep white matter	0.57 ± 0.73	0.70 ± 0.67	0.49

*Group differences between patients and controls were examined by using the χ^2^ test for sex and Welch’s t-test for age and Fazekas scales. The significance threshold was set at P < 0.05.

Because DDE is a relatively new technique for clinical research, we also examined the stability of the dMRI parameters in terms of scan–rescan repeatability in a separate group of 4 young healthy subjects (3 male and 1 female, 25–37 years old), who underwent two scans on different days within a week. In these subjects, we also acquired conventional SDE for comparison.

### Image Acquisition

The subjects were scanned by using a clinical 3T scanner (MAGNETOM Prisma, Siemens Healthcare, Erlangen, Germany) equipped with a 64-channel head coil using a prototype sequence. DDE data were acquired using a monopolar spin-echo type acquisition. We used parallel (LTE) and perpendicular (PTE) pairs of diffusion sensitizing gradient blocks, with 30 uniformly distributed directions. Note that, under the DTD assumption, the direction of PTE can be characterized by the normal vector. For both LTE and PTE, we used two shells, which had b-values of 1000 and 2000 s/mm^2^. One volume without diffusion weighting was also obtained. Thus, a total of 121 volumes were acquired. Other settings were image resolution = 3 × 3 × 3 mm^3^, TE = 93 ms, TR = 6300 ms, Δ = 18.3 ms, δ = 16.8 ms, mixing time = 24.9 ms, in-plane GRAPPA with acceleration factor 2, through-plane GRAPPA (SMS) with multiband factor 2, partial Fourier 6/8, 44 axial slices, and scan time 14 min. In addition, anatomical T1- and T2-weighted images were acquired and inspected by a neuroradiologist to rate Fazekas grade and to check for any other co-existing pathologies. SDE acquisition in the young subjects used the same b-values and 30 directions, resulting in a total of 61 volumes. The acquisition settings were identical to those of DDE, except Δ = 43.3 ms and δ = 36.4 ms. The scan time for SDE was 7 min.

### Image Processing

Images were pre-processed by using FSL 6.0.1 (Jenkinson et al., 2012) and MRTrix3 (Tournier et al., 2019). Raw images were denoised (Veraart et al., 2016) and corrected for Gibbs artifact (Kellner et al., 2016), eddy currents and motion (Andersson and Sotiropoulos, 2016), and B1 inhomogeneity (Tustison et al., 2010). For eddy-current correction of DDE, the second diffusion direction was used as the input to *eddy* in FSL (Yang et al., 2018). The dMRI parameter maps were computed using the multidimensional diffusion MRI toolbox (Nilsson et al., 2018). In a small number of voxels where the fitting resulted in negative values of *V*(*D^c^*), the fitting was repeated by using data smoothed with an isotropic 3D Gaussian kernel with sigma = 0.7 × voxel size.

### Stability of Measurements

Using the scan-rescan data from the 4 young subjects, we computed the within-subject coefficient of variation (CV_ws_), defined as ${\text{CV}}_{\text{ws}}=\frac{\sigma_{\text{ws}}}{\mu }\times 100\%$. Here, μ is the grand mean and σ_ws_ is the within-subject standard deviation. To compute CV_ws_ across the white-matter voxels, each subject’s FA map was non-linearly registered into the Johns Hopkins University (JHU) template in FSL. The other dMRI parameter maps were also transferred into the standard space by using the same deformation. A white-matter mask was generated by thresholding the FA template at FA > 0.2. To mitigate partial volume effects with the gray matter and cerebrospinal fluid (CSF), the mask was further eroded by one voxel. We also report CV_ws_ for the mean value of each white-matter region of interest (ROI) that was used for the analyses of aging and PD (Section “Statistical Analysis”).

### Statistical Analysis

**FIGURE 1:**
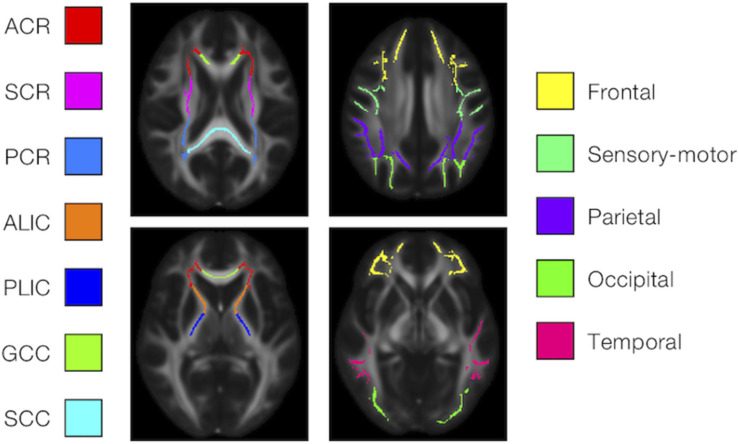
Atlas-based ROIs. Twelve ROIs were defined as the intersections between the TBSS-processed white-matter skeleton and the JHU and Harvard–Oxford atlases in FSL (ACR/SCR/PCR, anterior/superior/posterior corona radiata; ALIC/PLIC, anterior/posterior limb of the internal capsule; GCC/SCC, genu/splenium of the corpus callosum).

We examined the effects of the subjects’ characteristics (age, diagnosis, and UPDRS-III score) on the dMRI parameters by means of whole-brain voxel-wise analyses using tract-based spatial statistics (TBSS) (Smith et al., 2006) and atlas-based ROI analyses. First, all subjects’ FA images were aligned into a common space by means of non-linear registration, followed by creation of a mean FA skeleton. The threshold for creating the FA skeleton was set at FA > 0.2. Then, the aligned FA map of each subject was projected onto the FA skeleton. The other parameter maps were projected onto the mean FA skeleton by using the same transformation. Subsequently, the TBSS-processed skeletons were subjected to ROI analyses. Twelve white-matter ROIs were defined as the intersection of the white-matter skeleton and the JHU and Harvard–Oxford atlases in FSL as in Guerreri et al. (2019) (Figure 1), and the mean value within each ROI was extracted. Specifically, the deep white-matter ROIs (the anterior corona radiata [ACR], posterior corona radiata [PCR], superior corona radiata [SCR], anterior and posterior limb of the internal capsule [ALIC, PLIC], and genu and splenium of the corpus callosum [GCC, SCC]) were defined by using the JHU atlas, and the subcortical white-matter ROIs (frontal, sensory-motor, parietal, occipital, and temporal cortices) were defined by using the Harvard–Oxford atlas.

We opted not to exclude voxels with WMH from the analyses for the following two reasons. First, evidence suggests associations between WMH and the severity of motor and non-motor symptoms in PD (McDonald et al., 2016; Veselý et al., 2016). The pathophysiology of WMH is complex, and whether WMH are present in the patients because of incidental cerebrovascular disease or are a consequence of PD remains unclear (McDonald et al., 2016). Second, although TBSS allows the exclusion of voxels by using subject-specific lesion masks, this process causes differences in the degrees of freedom across voxels and may complicate the interpretation of statistical results (Benitez et al., 2018).

For both the TBSS and ROI-based analyses, we fit linear models where the dependent variable was each dMRI parameter. First, we examined the effect of age in the healthy subjects, using sex as a nuisance covariate. Then, we examined the differences between the healthy subjects and the patients with PD, using age and sex as nuisance covariates. Finally, we examined the correlation with UPDRS-III score in the patients, using age and sex as nuisance covariates. Because motor impairment might be correlated with age, we checked for multi-collinearity using the variance inflating factor (VIF) (Hair et al., 2010). Although we used a stringent criterion of VIF < 4.0 (Hair et al., 2010), VIF was below this threshold for all predictors in the linear models. Statistical inference of the linear models was conducted by using permutation analysis (Winkler et al., 2014). For computational efficiency, we used 2000 permutation and tail approximations (Winkler et al., 2016). For TBSS, threshold-free cluster enhancement (Smith and Nichols, 2009) was applied. The significance threshold was set at *P* < 0.05, corrected for family-wise error (FWE) for multiple comparisons across voxels or ROIs. For the ROI-based analyses, we also report results without FWE correction (*P*_uncorrected_ < 0.05) because these data may be informative for future studies with larger samples.

## Results

### Scan-Rescan Repeatability

**FIGURE 2:**
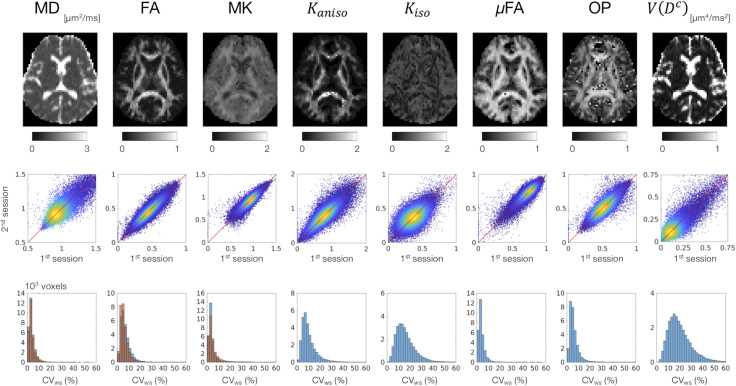
Scan–rescan repeatability of the dMRI parameters derived from DDE. Top: the diffusion parameter maps from a representative subject. Middle: scatterplots comparing the first and the second sessions. Values from the white-matter voxels of all 4 subjects are shown. Warmer color represents higher density of that point. Bottom: within-subject coefficient of variation (CV_ws_) in the white-matter voxels. The blue and orange histograms represent CV_ws_ of the dMRI parameters derived from DDE and SDE, respectively.

MD, FA, and MK showed excellent repeatability, with CV_ws_ below 6–8% for most of the white-matter voxels (Figure 2). The values of CV_ws_ were similar between DDE and SDE. μFA and OP also showed CV_ws_ below 8% for most of the voxels. The parameters of size variance [*K*_*iso*_ and *V*(*D^c^*)] showed greater CV_ws_, falling in the range of 10–25% for most of the voxels. As for ROI-based measurements, CV_ws_ was below 2% in all ROIs for MD, FA, μFA, and OP (Table 2). MK and *K*_*aniso*_ exhibited slightly greater values of CV_ws_ ranging from 0.5 to 4.8%. Although *K*_*iso*_ and *V*(*D^c^*) showed CV_ws_ below 6% for most of the ROIs, poorer repeatability was found in the internal capsule and corpus callosum with CV_ws_ around 10%, reflecting the challenges of dMRI in these regions due to partial volume effects with the CSF and the gray matter, Gibbs artifact, and CSF pulsation.

**TABLE 2:** Within-subject coefficients of variation of the ROI means (values are percentages).

ROIs	MD	FA	MK	*K*_aniso_	*K*_iso_	*μ*FA	OP	*V*(*D*^c^)	MD_SDE_	FA_SDE_	MK_SDE_
Frontal	0.5	1.1	1.3	2.1	0.8	0.6	0.8	1.4	1.1	3.2	0.7
Sensory- motor	0.5	0.8	0.5	1.8	1.9	0.5	0.9	2.9	0.4	1.4	0.6
Parietal	0.4	0.7	0.6	2.6	3.0	0.9	0.9	3.1	0.4	0.8	0.8
Occipital	0.6	1.1	1.0	3.0	3.9	1.0	0.9	4.2	0.5	0.6	0.8
Temporal	0.6	1.0	0.7	3.3	3.3	1.5	1.0	3.6	0.6	2.5	1.5
ALIC	0.9	1.1	3.4	2.3	9.8	1.0	1.3	10.3	0.6	1.6	2.0
PLIC	0.6	0.8	1.7	1.7	6.1	0.5	0.4	6.4	1.4	1.9	1.2
ACR	1.1	1.6	1.3	3.6	3.5	1.2	0.5	4.5	0.7	1.5	1.0
SCR	0.4	1.3	1.0	2.1	3.2	0.7	0.7	3.5	0.8	1.1	1.0
PCR	0.6	0.9	1.2	2.2	1.7	0.7	0.5	2.2	1.4	0.9	0.7
GCC	0.7	1.2	4.8	4.2	10.9	0.9	0.3	7.5	3.9	2.7	2.4
SCC	0.5	0.6	1.2	2.2	4.0	0.4	0.7	3.4	2.5	1.1	2.1

*ACR/SCR/PCR, anterior/superior/posterior corona radiata; ALIC/PLIC, anterior/posterior limb of the internal capsule; GCC/SCC, genu/splenium of the corpus callosum.*

### Correlation With Age in the Healthy Subjects

**FIGURE 3:**
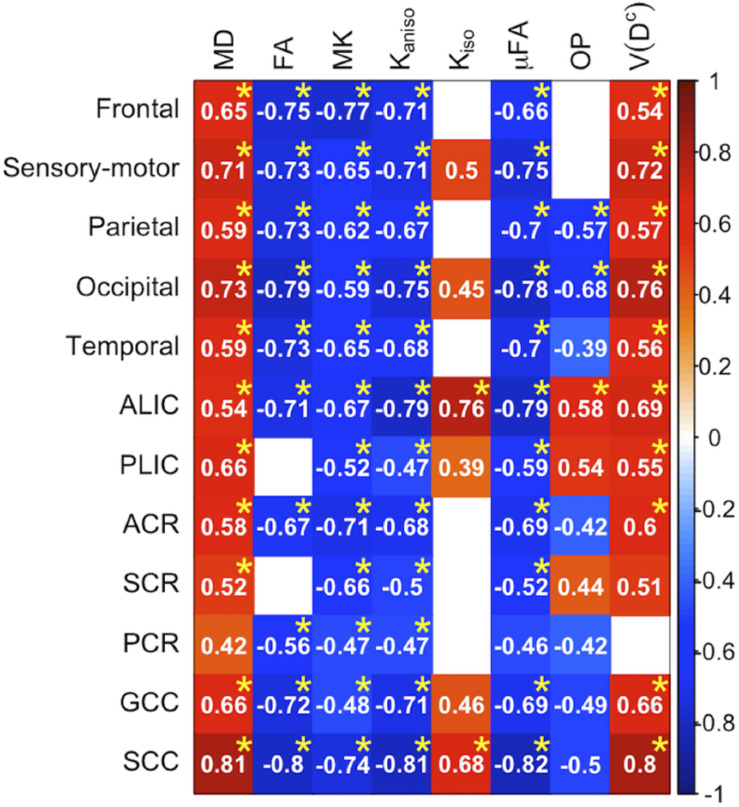
Correlation with age in the healthy control subjects. Pearson’s correlation coefficients are shown where a correlation between a diffusion parameter and age was found (*P*_uncorrected_ < 0.05). Red denotes positive correlation; blue indicates negative correlation. Asterisks indicate significant correlations after FWE correction. ACR/SCR/PCR, anterior/superior/posterior corona radiata; ALIC/PLIC, anterior/posterior limb of the internal capsule; GCC/SCC, genu/splenium of the corpus callosum.

**FIGURE 4:**
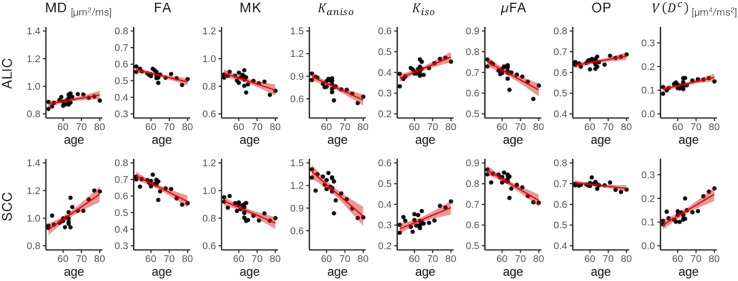
Correlation with age in two representative ROIs. Red lines represent linear fitting where a significant correlation was found (*P*_uncorrected_ < 0.05). The shaded area represents the 95% confidence interval. ALIC, anterior limb of the internal capsule; SCC, splenium of the corpus callosum.

**FIGURE 5:**
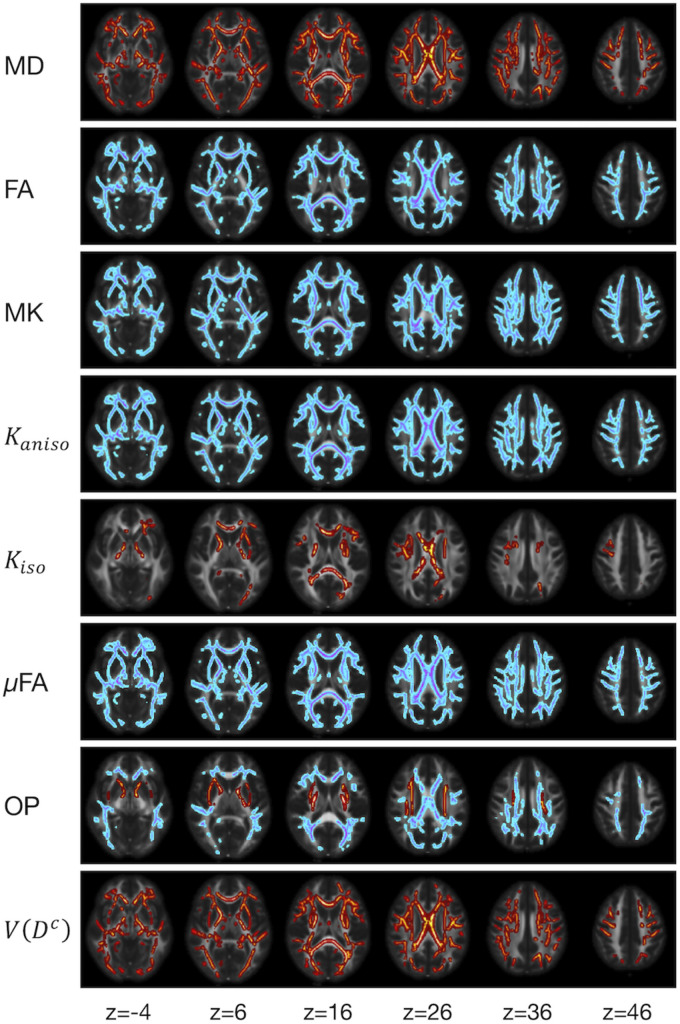
TBSS results for correlation with age in the healthy control subjects. Voxels with significant correlation after the FWE correction (*P* < 0.05) are shown. Hot colors denote positive correlation, whereas cold colors denote negative correlation. To aid visualization, the results were thickened by using the *fill* function in FSL.

To display the results of ROI-based correlations, we adopted a figure format used in Billiet et al. (2015) and Guerreri et al. (2019) (Figure 3). For MD and *V*(*D^c^*), positive correlation with age was seen in extensive regions of the white matter in both ROI-based analyses and TBSS (Figures 3–5). FA, MK, *K*_*aniso*_, and μFA showed negative correlation with age within these regions. OP demonstrated a unique behavior in that both positive and negative correlations were observed, depending on anatomical locations. In particular, although the correlation was negative in most of the white-matter regions, positive correlation was observed in the ALIC and PLIC, external capsule, and SCR. *K*_*iso*_ showed positive correlation with age, although in fewer regions compared with the other diffusion parameters.

### Difference Between the Healthy Control and the Patients With PD

**FIGURE 6:**
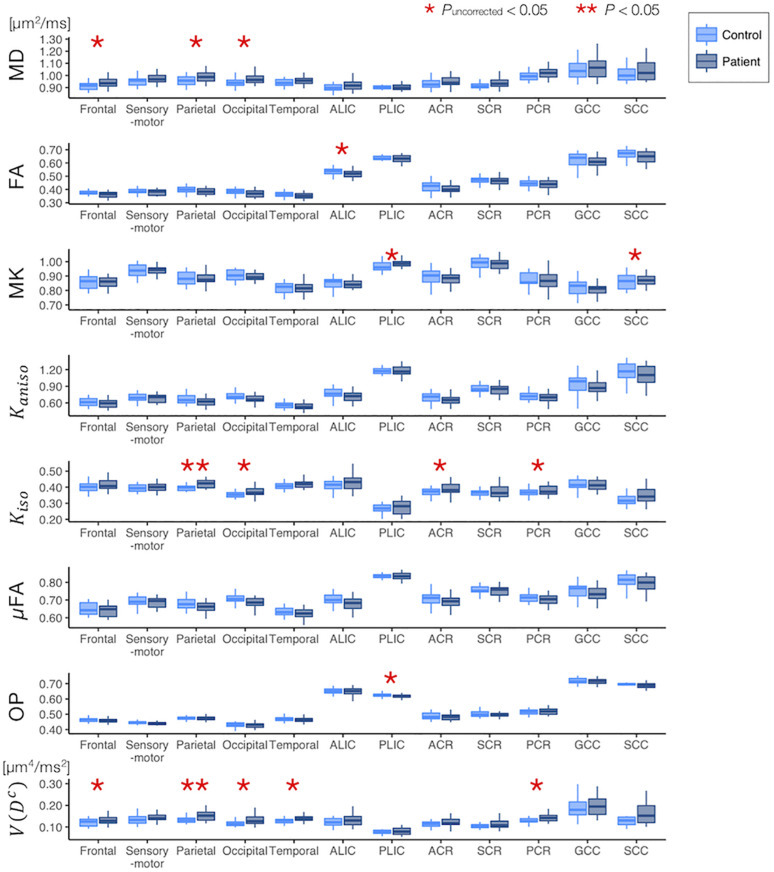
Differences between the healthy control subjects (light blue) and the patients with PD (dark blue) in the ROI-based analyses. The red asterisks denote statistically significant differences (*P_uncorrected_ < 0.05, **P < 0.05). ACR/SCR/PCR, anterior/superior/posterior corona radiata; ALIC/PLIC, anterior/posterior limb of the internal capsule; GCC/SCC, genu/splenium of the corpus callosum.

**FIGURE 7:**
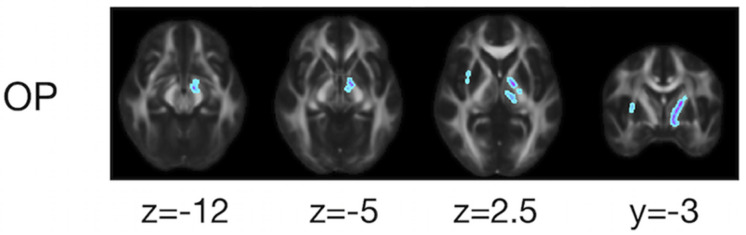
TBSS results for group differences between the healthy control subjects and the patients with PD. Voxels with significant differences after the FWE correction (*P* < 0.05) are shown. Voxels where the patients exhibited smaller values are represented in cold colors. In no voxels did patients exhibit greater values. To aid visualization, the results were thickened by using the *fill* function in FSL.

The ROI-based analyses revealed that the patients with PD had greater values of MD, *K*_*iso*_, and *V*(*D^c^*) in the subcortical white-matter regions than the controls (Figure 6), although only the differences in *K*_*iso*_ and *V*(*D^c^*) of the parietal ROI remained significant after the FWE correction. The group differences were below the significance threshold for *K*_*aniso*_ and μFA for all ROIs. Slightly greater values of MK were observed in the patients in the PLIC and SCC. Smaller values of FA and OP were observed in the ALIC and PLIC, respectively. In TBSS, only OP revealed statistically significant results: the patients exhibited smaller values in the left internal capsule, the left cerebral peduncle, the left thalamus, and the right external capsule (Figure 7).

### Correlation With Motor Impairment in PD

**FIGURE 8:**
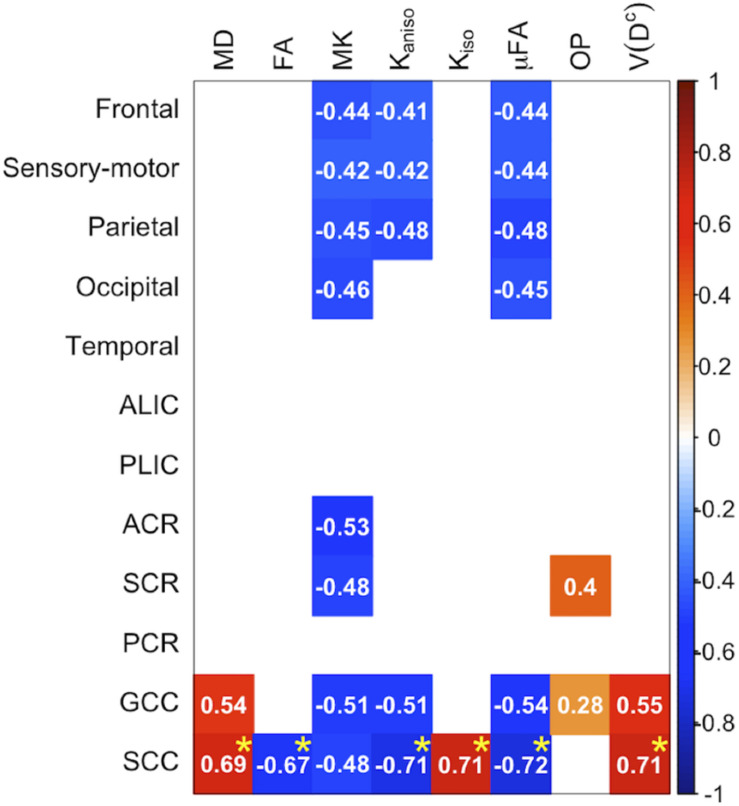
Correlation with UPDRS-III score in the patients with PD. The Pearson correlation coefficients shown indicate significant correlation between a diffusion parameter and UPDRS-III score (*P*_uncorrected_ < 0.05). Red denotes positive correlations; blue indicates negative correlations. Asterisks indicate significant correlations after FWE correction. ACR/SCR/PCR, anterior/superior/posterior corona radiata; ALIC/PLIC, anterior/posterior limb of the internal capsule; GCC/SCC, genu/splenium of the corpus callosum.

**FIGURE 9:**
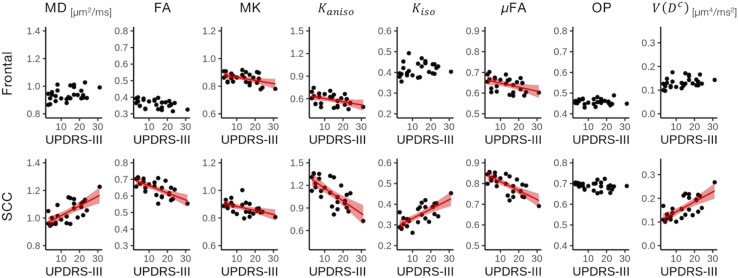
Correlation with UPDRS-III score in two representative ROIs. Red lines represent linear fitting where significant correlation was found (*P*_uncorrected_ < 0.05). The shaded area represents the 95% confidence interval. SCC, splenium of the corpus callosum.

**FIGURE 10:**
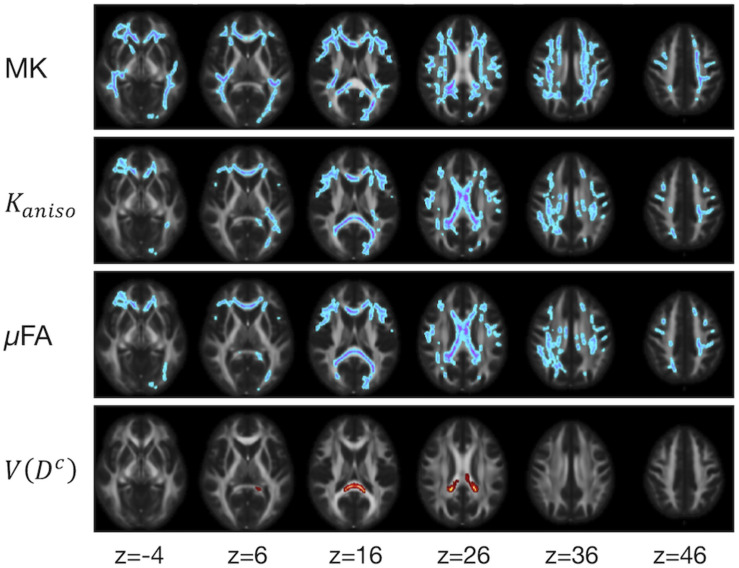
TBSS results for correlation with UPDRS-III score in the patients with PD. Voxels with significant correlation after the FWE correction (*P* < 0.05) are shown. Hot colors denote positive correlation, whereas cold colors denote negative correlation. To aid visualization, the results were thickened by using the *fill* function in FSL.

The ROI-based analyses revealed positive correlations with UPDRS-III score for MD, *K*_*iso*_, and *V*(*D^c^*), and negative correlations for FA, MK, *K*_*aniso*_, and μFA in GCC and SCC (Figures 8, 9). MK, *K*_*aniso*_, and μFA exhibited negative correlations also in the subcortical white-matter ROIs. TBSS showed negative correlations for MK, *K*_*aniso*_, and μFA in extensive areas in the frontal and parietal lobes and the corpus callosum, and positive correlation for *V*(*D^c^*) in SCC (Figure 10). For MD, FA, and OP, the correlations were below the significance threshold in TBSS.

## Discussion

This study investigated the utility of DTD parameters derived from DDE to characterize white-matter degeneration in aging and PD. Advanced age was associated with greater MD and smaller FA, MK, *K*_*aniso*_, and μFA, in agreement with previous studies that used SDE (Madden et al., 2012; Coutu et al., 2014; Billiet et al., 2015; Benitez et al., 2018; Guerreri et al., 2019) and DDE (Lawrenz et al., 2016). OP decreased with age for most of the voxels, which, together with the decrease of μFA, led to the age-related decrease of FA. At the same time, a positive correlation between OP and age was observed in some white matter regions including the ALIC, PLIC, and SCR. Our results of age-related changes in μFA and OP replicated the findings by the earlier study (Lawrenz et al., 2016). Age-related OP increase in some regions can be understood as selective degeneration of the secondary crossing fibers, thus increasing the proportion of aligned fibers. The work by Lawrenz et al. (2016) employed a different approach than the DTD model, i.e., microscopic anisotropy estimation proposed in Lawrenz et al. (2010) which is based on the signal equation of restricted diffusion provided by Mitra (1995). Though it is beyond the scope of this study to examine which model should be preferred, it seems that the observed trends in age-related changes of μFA and OP are robust against this difference in model assumptions. We also found an age-related increase in the measures of size variance [*K*_*iso*_ and *V*(*D^c^*)]. The effects of aging and PD were in the same directions for most of the diffusion parameters and anatomical locations, such as increases of MD, *K*_*iso*_, and *V*(*D^c^*) and decreases of FA, *K*_*aniso*_, and μFA. This is biologically plausible, because neurodegeneration in aging and PD have many features in common, including a declined ability to homeostatically regulate proteostasis, neuroinflammation, mitochondrial dysfunction, oxidative stress, degeneration of the myelin sheath, accumulation of cellular debris, alteration of axonal transport, axonal swelling/beading, and loss of axons (Tagliaferro et al., 2015; Collier et al., 2017; Salvadores et al., 2017; Calabrese et al., 2018; Datar et al., 2019). The present results also suggest that the measures of microscopic anisotropy (*K*_*aniso*_ and μFA) might be useful to track white-matter degeneration related to the motor impairment in PD.

Through the analysis of underlying kurtosis sources via the DTD model, we found that the reductions of MK reported in aging (Coutu et al., 2014; Benitez et al., 2018; Guerreri et al., 2019) and PD (Kamagata et al., 2013, 2014) are likely driven by the reduction of microscopic anisotropy. The decomposition of kurtosis sources (Eq. 12) suggests that either an increase of *K*_*iso*_ or a decrease of OP would lead to an increase of MK. We indeed observed slightly greater MK in the PLIC and SCC in the patients than in the controls. Although this appears to contradict previous reports of smaller MK in patients (Kamagata et al., 2013, 2014), it may instead indicate that the increase of *K*_*iso*_ and decrease of *K*_*aniso*_ have different time trajectories during disease progression. If the increase of *K*_*iso*_ is related to early neuroinflammation and the decrease of *K*_*aniso*_ represents the subsequent degeneration, as speculated by Andica et al. (2019a), MK may possibly have a non-monotonical trajectory, with an initial increase followed by decrease, assuming constant OP (Eq. 12). Future studies to investigate the trajectories of the DTD parameters in relation to disease progression are warranted. We also demonstrated that microscopic anisotropy can be estimated with excellent repeatability comparable to that of SDE-derived DTI/DKI parameters in both the present study and the literature (Shahim et al., 2017; Ades-Aron et al., 2018). Taken together, the present results are encouraging that multidimensional diffusion encoding could be implemented clinically. Although we used a relatively long acquisition for this exploratory study, μFA can be estimated reliably with a reduced DDE acquisition (Yang et al., 2018; Kerkelä et al., 2020) to make the scan time clinically feasible.

Our results support several hypothetical interpretations of previous SDE studies that relied on more restrictive assumptions. (Chad et al., 2018) and (Andica et al., 2019a) applied regularized fitting of the bi-tensor free-water DTI model (Pasternak et al., 2009) to study aging and PD, respectively. Their results showed that aging and PD are associated with an increase of the free-water fraction in the white matter. A larger free-water fraction would lead to greater size variance and smaller microscopic anisotropy (Szczepankiewicz et al., 2015; Westin et al., 2016), as observed in our study. Certainly, an increase in free water is not the only possible mechanism, and further investigation is required to elucidate specific pathological features. For example, patchy axonal loss and demyelination may lead to similar results via heterogenous increase of the radial diffusivities of compartmental diffusion tensors. Changes in axon morphology such as swelling and beading, which would reduce axial diffusivities of compartmental diffusion tensors (Budde and Frank, 2010; Palombo et al., 2018) and hence μFA, also occur heterogeneously within a voxel and may lead to an increase of size variance. (Ikenouchi et al., 2020) estimated μFA by using SDE under the assumption of uniform size and shape of compartmental diffusion tensors (Kaden et al., 2016) and reported a reduction of μFA in patients with PD and correlation with UPDRS-III score, in line with our observations. Although the methods of Pasternak et al. (2009) and Kaden et al. (2016) were designed to be practical for use with clinical SDE data, the dependence on regularization or strongly restrictive assumptions may bias the output parameters (Molina-Romero et al., 2018; Henriques et al., 2019) and therefore confirmation with extended acquisition, as we used in the current study, is important.

Although the differences between the controls and the patients were less prominent than the effects of age, smaller OP in the patients was identified in the left cerebral peduncle and left internal capsule (Figure 7). This overlaps the anatomical location of the medial forebrain bundle (Coenen et al., 2018), a fiber bundle that is involved early in PD (Tagliaferro et al., 2015). Our results are in line with those of previous studies that observed reduced FA within this region (Planetta et al., 2013; Zhang et al., 2015), as well as with reports of left-predominant involvement (Prakash et al., 2012; Scherfler et al., 2012).

Interestingly, we observed *K*_*aniso*_ and μFA were not very sensitive to the group differences between the normal subjects and the patients with PD (Figure 6), despite their correlation with the motor impairment. In contrast, MD, *K*_*iso*_, and *V*(*D^c^*) showed some sensitivity to the group differences in several ROIs but their correlation with motor impairment was limited to the callosal ROIs (Figure 8). Although the mechanisms behind these observations remain unknown, our speculation is that these two groups of diffusion parameters weigh different facets of neurodegeneration. It has been hypothesized that age-related changes form pathological foundation on which PD-related neurodegeneration build (Collier et al., 2017), and that there is a biological interaction between aging and PD (Levy, 2007). We speculate that microscopic anisotropy has relatively greater weight on the accumulation of age-related neuronal damage that is perhaps accelerated in PD and exacerbates the clinical expression of symptoms, while reduced microscopic anisotropy is less specific to the diagnosis of PD. On the other hand, MD, *K*_*iso*_, and *V*(*D^c^*) may be influenced more by the factors that differentiate PD from normal aging but are not proportional to disease progression.

Several possible confounding effects with regard to acquisition and parameter estimation need to be mentioned. First, by adopting the DTD model, which does not feature the effects of timing parameters (diffusion gradient duration, separation between the gradients, and the mixing time), we assumed the effects of timing parameters were negligible. Though this has some support by earlier studies observing the effects of diffusion time were small in brain tissue for the range above 10 ms (Clark et al., 2001; Portnoy et al., 2013), more recent studies suggest observable time-dependence in the human white matter for even longer times (Fieremans et al., 2016; Lee et al., 2020). A recent observation of intra-compartmental kurtosis in mouse brains (Henriques et al., 2020) also indicate the contribution of restricted compartments might be non-negligible. Taken together, the DTD model employed in this study may not fully capture the microstructural complexities and a more adequate model would need to feature the effects that explain the reported time-dependence, like restricted compartments (Yolcu et al., 2016; Özarslan et al., 2017), structural disorder (Novikov et al., 2014), or inter-compartmental exchange (Nilsson et al., 2013). Incorporating the timing parameters as additional measurement dimensions of DDE is expected to identify a suitable extension of the model in future. Though dependence on the DTD model is an essential limitation to our study, in Kerkelä et al. (2020), μFA estimated under this assumption approximated the gold-standard model-free method (Ianuş et al., 2018) very well in human white matter. Therefore, we believe that any bias introduced by the model assumption was not large, at least for μFA and *K*_*aniso*_. Second, the expansion Eq. 2 is valid only in the vicinity of *b* = 0 (Westin et al., 2016; Topgaard, 2017), as in the case of DTI/DKI (Kiselev, 2013). Although the covariance tensor framework is computationally efficient, a possible bias in case of finite signal-to-noise ratio has been pointed out (Reymbaut et al., 2020). In addition, our scan–rescan analyses revealed problematic instability of the *V*(*D^c^*) and *K*_*iso*_ estimation, which needs to be addressed before clinical application of these metrics. Third, the acquisition used in this study was not controlled for the concomitant fields (transverse magnetic field components accompanying the applied gradient to satisfy Maxwell’s equations) (Baron et al., 2012), which cause bias in the signal and hence the DTD parameters (Szczepankiewicz et al., 2019). This issue would be mitigated by the use of multiple refocusing pulses to make the diffusion encoding symmetric (Callaghan and Komlosh, 2002).

Our study has several limitations. First, PD is a heterogenous disease whose clinical manifestations vary widely among individual patients (Fereshtehnejad et al., 2017). Robust determination of PD subtypes based on cross-sectional data remains a challenge (Simuni et al., 2016) and needs to be addressed in future work. Also, though we merged the left and right ROIs for this exploratory study, neurodegeneration in PD has some asymmetry which correlates with the laterality of symptoms (Riederer et al., 2018). Further investigation with a large sample will be necessary to elucidate the relationships between white-matter degeneration and detailed clinical features, including both motor and non-motor symptoms. Second, we did not prove clinical benefit of DDE as compared to the standard SDE. Though μFA showed better correlation with age and motor impairment than FA, similar correlation could be observed with MK. Given the longer scan time of DDE, DTI/DKI based on standard SDE is still a powerful option for clinical studies. However, the strength of this study is that we showed the sensitivity of MK to neurodegeneration is likely attributed to its link to microscopic anisotropy. Such knowledge can be informative for future researches in selecting dMRI acquisition and parameter to study particular diseases. Third, we applied simplistic linear models for statistical analyses, but the effects of age might differ between the patients and the controls (Zhang et al., 2016; Pozorski et al., 2018). Also, the effect of PD might differ between males and females (Gillies et al., 2014). Although adding age × disease and sex × disease interactions in the linear model did not reveal any significant interactions for the present data (not shown), several imaging studies have suggested that these interactions are non-negligible (Dean et al., 2016; Yadav et al., 2016). The absence of these interaction effects in the present study may be attributed to the relatively small sample size which was not optimally matched regarding male/female ratio. Fourth, the majority of patients had relatively long disease duration and were already under medication, and therefore we could not quantify the effects of medication (Atkinson-Clement et al., 2017) and/or the non-linear (U-shaped) trajectories presumably related to neuronal compensation (Sanjari Moghaddam et al., 2020). Lastly, the scan–rescan repeatability should ideally be measured in a cohort similar to the patients because motion artifacts might be severer in these subjects than in young and healthy subjects. Also, the within-subject variability needs to be evaluated relative to the between-subject variability in the patients.

## Conclusion

In this study, we explored the utility of DDE-derived parameters for characterizing white-matter degeneration in aging and PD. Advanced age was associated with greater mean size and size variance of the compartmental diffusion tensors and with smaller microscopic anisotropy. We found the reductions of MK in aging and PD reported in the literature are likely driven by the reduction of microscopic anisotropy. Furthermore, microscopic anisotropy correlated with the severity of motor impairment in PD. We further showed that microscopic anisotropy can be estimated with excellent repeatability by using modern clinical scanners. In conclusion, multidimensional diffusion encoding can provide more comprehensive and clinically relevant information about the white-matter degeneration in aging and PD than conventional SDE-based methods.

## Data Availability Statement

The raw data supporting the conclusions of this article will be made available by the authors, without undue reservation, to any qualified researcher.

## Ethics Statement

The studies involving human participants were reviewed and approved by the Institutional Review Board of Juntendo University School Hospital. The patients/participants provided their written informed consent to participate in this study.

## Author Contributions

KKami and KKama contributed to conception and design of the study. KKama, KO, TH, TO, and HT-A organized the data acquisition and quality control. KKami, SM, and CA performed the image processing. KKami performed the statistical analysis and wrote the first draft of the manuscript. KM, TF, MH, NH, and SA revised the subsections of the manuscript. All authors contributed to the final manuscript revision, read and approved the submitted version.

## Conflict of Interest

KM is an employee of Siemens Healthcare K.K. TF is an employee of Siemens Healthcare GmbH. The remaining authors declare that the research was conducted in the absence of any commercial or financial relationships that could be construed as a potential conflict of interest.
